# Risk of dry eye syndrome after radiotherapy for orbital tumors: a systematic review and meta-analysis

**DOI:** 10.3389/fonc.2026.1786414

**Published:** 2026-03-25

**Authors:** Mengyu Zong, Changyong Li

**Affiliations:** Institute of Rehabilitation Medicine, Qilu Medical University, Zibo, Shandong, China

**Keywords:** dry eye syndrome, meta-analysis, orbital tumor, radiotherapy, systematic review

## Abstract

**Background:**

This systematic review aimed to investigate the risk of developing dry eye syndrome following radiotherapy for orbital tumors. This study aimed to provide insights for formulating radiotherapy suggestions for orbital tumors and preventing dry eye syndrome.

**Methods:**

This systematic review collected literature published in PubMed, EBSCO, and Web of Science prior to December 1,2025 concerning dry eye syndrome induced by radiotherapy for orbital tumors. 23 studies total 1,602 patients with orbital tumors were included across all studies. The review adhered to the PRISMA guidelines, with literature quality assessed using the Newcastle-Ottawa Scale (NOS) scale. All data analyses were conducted using Stata 17.

**Results:**

The overall prevalence of Dry Eye Syndrome (DES) after orbital radiotherapy was 31% (95% CI: 15–48%, P< 0.01), with high heterogeneity (I^2^ = 98.56%). Subgroup analyses indicated higher DES incidence in patients aged ≥60 years (41% vs. 28%), those receiving ≥30 Gy (36% vs. 25%), and those with non-orbital lymphoma (39% vs. 29%). Longer follow-up (≥5 years) was associated with lower incidence (19% vs. 40%). No significant publication bias was detected.

**Conclusion:**

Radiotherapy for orbital tumors significantly increases the risk of DES. Although statistical interactions were not significant, trends suggest that older age, higher radiation dose, and shorter follow-up may elevate DES risk, underscoring the need for tailored monitoring and preventive care.

**Systematic review registration:**

https://www.crd.york.ac.uk/prospero/, identifier.

## Introduction

Orbital tumors are diseases that can compromise visual function, facial appearance, and even life itself, primarily encompassing benign and malignant tumors. Benign tumors are chiefly represented by orbital pseudotumors, while malignant tumors include orbital lymphoma and orbital rhabdomyosarcoma. Providing effective treatment protocols is particularly crucial for orbital diseases, especially malignant orbital tumors. Surgical intervention alone often fails to achieve curative outcomes. Within comprehensive treatment strategies, radiotherapy has evolved into a specialized discipline, playing a vital role in the management of orbital tumors and emerging as a primary adjunctive modality (1–4). Radiotherapy protocols vary according to tumor subtype and radiosensitivity; for instance, orbital mucosa-associated lymphoid tissue lymphoma primarily requires low-dose radiotherapy (5, 6). Consequently, increasing attention is being directed towards the application of radiotherapy in ocular and orbital diseases.

While ionizing radiation offers therapeutic benefits for ocular tumors, certain highly radiosensitive ocular structures—such as the lacrimal glands and meibomian glands—may sustain permanent loss of secretory function, leading to irreversible dry eye syndrome. Studies have summarized acute and chronic toxic reactions to low-dose orbital lymphoma radiotherapy, with 6–42% of patients experiencing grade 1 toxic effects such as dry eye syndrome. The probability of toxic reactions occurring with medium-dose radiation reaches up to 96% (7, 8).

Although several studies have reported DES following orbital radiotherapy, a comprehensive synthesis of evidence regarding its incidence and influencing factors is lacking. This systematic review and meta-analysis aimed to quantify the risk of DES after radiotherapy for orbital tumors and explore potential moderators such as age, radiation dose, and tumor subtype.

## Methods

### Search strategy

This review collates literature published in PubMed, EBSCO, and Web of Science prior to December 2025 concerning radiation-induced dry eye syndrome in orbital tumors. The specific search strategy employed was as follows:(“ocular adnexal lymphoma “or “MALTomas” or “lymphoma” or “orbital lymphoma”or”orbital pseudotumor”or “tumor”or “risk” or “effect”)and(“dry eye syndrome” or “dry eye disease” or “dry eye” or “ ocular complications” or “ocular “ or “meibomian glands”)and (“radiotherapies” or “radiation therapy” or “radiation therapies” or “radiation treatment” or “radiotherapy, targeted” or “targeted radiation therapy” or “therapy, targeted radiation” or “radiation” or “irradiation”). P (Population): Patients diagnosed with orbital neoplasms/tumors. I(Intervention): Radiotherapy to the orbital region (including X-rays and proton beam therapy) in combination with chemotherapy. C (Comparison): Baseline status of the patients before treatment (Pre-treatment vs. Post-treatment). O (Outcome): Incidence or severity of (DES), evaluated by clinical diagnostic criteria or patient-reported symptom scales. S (Study Design): Observational studies, including cross-sectional, case-control, and prospective or retrospective cohorts.

This review has been registered in the Prospective Systematic Reviews Register established collaboratively by the Centre for Evaluation and Dissemination of Health Research and Innovation at the UK National Institute for Health Research, with registration number CRD420251266569.

### Inclusion criteria

Following the search, each reference was screened individually to eliminate duplicates. Only English-language literature was included as a retrieval criterion, focusing on the fields of medicine and radiotherapy. References were screened based on article titles and abstracts. Studies addressing dry eye syndrome following radiotherapy for orbital tumors were included, provided they were original articles published in indexed journals. For the purpose of this review, DES was identified based on the diagnostic criteria reported in the original studies, which typically included objective clinical assessments (Schirmer test<10 mm, TBUT<5 s) and/or subjective symptom scales (OSDI >12). Studies were included if they provided a diagnosis of DES or reported relevant ocular surface parameters, even if not all three metrics were simultaneously present.

### Exclusion criteria

Subsequently, literature exclusion was conducted, excluding studies where full-text was unavailable, non-English publications, review articles, animal radiotherapy experiments, and radiotherapy research on head and neck cancers other than orbital tumors. Based on the search results, other papers related to head and neck tumors were excluded. Screening criteria were applied by two reviewers; in the event of disagreement during this process, the two individuals shall jointly deliberate and make the final decision.

**Figure 1:**
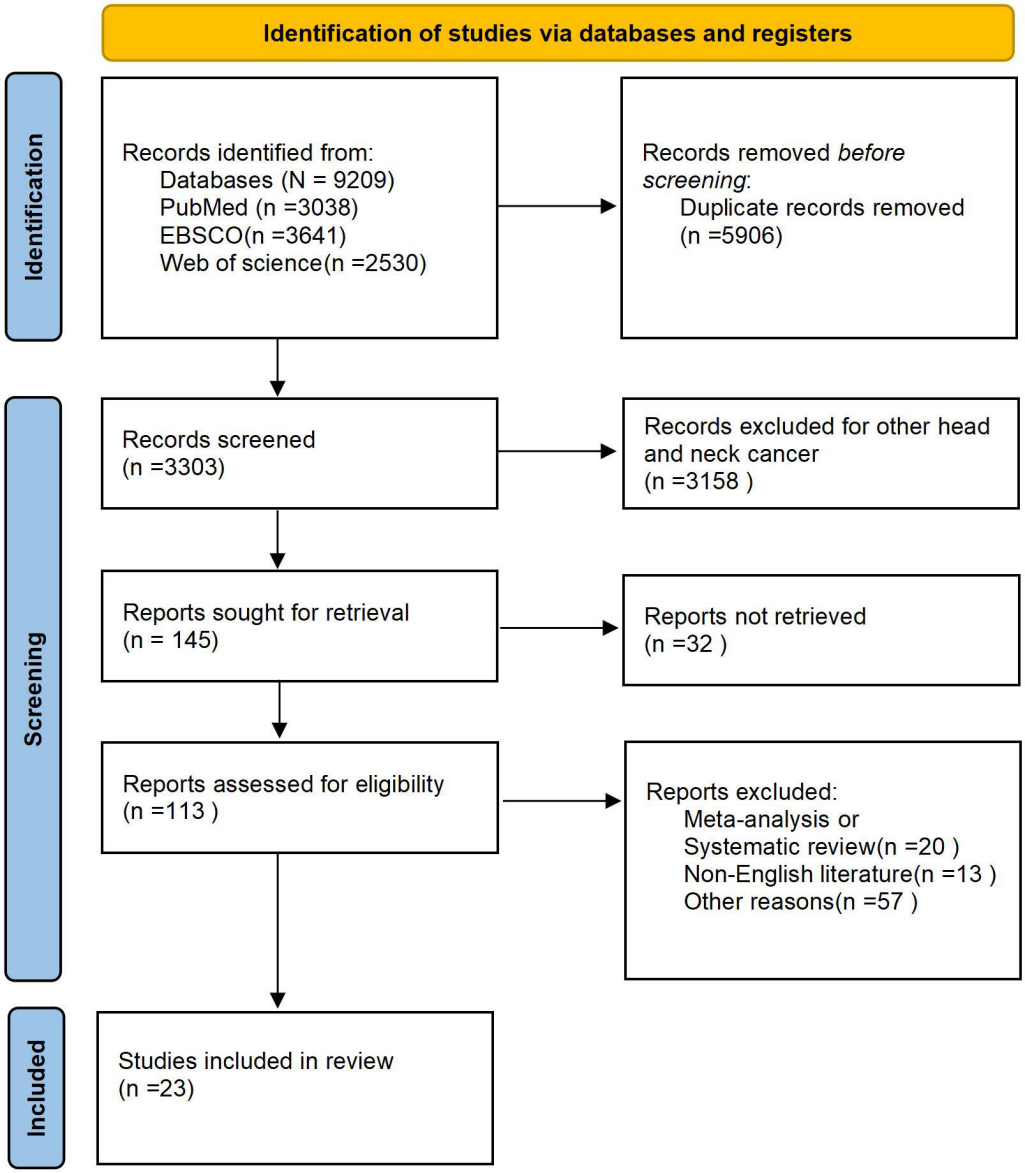
Flowchart of the literature screening process for this analysis.

The specific retrieval process followed the PRISMA flow diagram, as illustrated in (**Figure 1**). Data extraction from studies is summarized (**Table 1**). Standardized data sheets were used to collect the following information: 1. First author’s name; 2. Publication year; 3. Study design; 4. Number of patients and median age; 5. Orbital tumor type/histological type; 6. Incidence/grade of dry eye syndrome; 7. Radiotherapy dose/dose rate; 8. Outcomes and follow-up duration.

**Table 1:** Analysis of studies on radiation-induced dry eye syndrome in patients with orbital tumors.

Author	Year	Article design	Sample size	Age	Types of orbital tumors/histological types	Prevalence/severity grading of dry eye	Radiotherapy dose/dose rate	Follow-up
Raffaele Nuzzi (9)	2020	Retrospective analysis	8	/	Orbital lymphoma	24 Gy irradiation was 66.67%, 4 Gy irradiation was 50%.	Six cases 24 Gy, 2 Gy per day; Two cases 4 Gy, 2 Gy per day.	45.67 months.
NAOKI HAS-HIMOTO (10)	2012	Retrospective analysis	78	60	Patients with stage I MALT lymphoma of the ocular adnexa	Three cases developed Grade 2 dry eye complications, and one case developed Grade 3 dry eye complications.	The median radiotherapy dose was 30.6 Gy.	66 months.
Olga Esik (11)	1996	Retrospective analysis	37	56	Extraorbital non-Hodgkin lymphoma	One case of severe dry eye syndrome	The median radiotherapy dose was 40 Gy.	91.2 months.
Roshan S. Prabhu (12)	2012	Retrospective analysis	20	/	Pseudotumor of the orbit	1 case of chronic dry eye syndrome	The median radiotherapy dose was 27 Gy.	18.6 months.
Carolina E. Fasola (13)	2013	Retrospective analysis	20	/	Extraorbital non-Hodgkin lymphoma	1 case of dry eye syndrome	Total dose: 4 Gy, fractionated at 2 Gy per fraction	26 months.
Yasuo Ejima (14)	2006	Retrospective analysis	42	61	Ocular adnexal lymphoma	41 cases of dry eye	The median radiotherapy dose for 21 cases was 30.6 Gy, while the median radiotherapy dose for the remaining 21 cases was 30 Gy.	48 months.
Jayant S. Goda (15)	2010	Retrospective analysis	71	/	Orbital lymphoma	2 cases of dry eye	The median radiotherapy dose was 25 Gy.	88.8 months.
Christian Hoffmann (16)	2022	Retrospective analysis	62	66	Orbital lymphoma	21.2% cases of dry eye	The median radiotherapy dose was 30.6 Gy.	43.5 months.
Shubha Tiwari (17)	2017	Retrospective analysis	47	39.8	Orbital radiotherapy	47.07% cases of dry eye	The median radiotherapy dose was 48.99 Gy.	18.6 months.
Jayant Sastri Goda (18)	2011	Retrospective analysis	89	56	Orbital lymphoma	22 cases of dry eye	Ninety-seven per cent of patients received a dose of 25 Gy, while three per cent received a dose of 30 Gy.	70.8 months.
R. Beverly Raney (19)	2000	Retrospective analysis	94	/	Orbital rhinoblastoma	24 cases of dry eye syndrome	Group II: 41.4 Gy Group III: 41.4 Gy–50.4 Gy	91.2 months.
Quynh-Thu Le (20)	2002	Retrospective analysis	40	54	Orbital lymphoma	5 cases of dry eye	The median radiotherapy dose was34 Gy.	70.8 months.
PING ZHOU (21)	2005	Retrospective analysis	46	59	Orbital lymphoma	30% cases of dry eye	The median radiotherapy dose was 30.6Gy.	46 months.
Pereira-Da Silva (22)	2023	Retrospective analysis	149	/	Lymphoma of the orbit and adnexa	16% cases of dry eye	The median radiotherapy dose was 25 Gy.	73.2 months.
Sanjna Shelukar (23)	2022	Retrospective analysis	17	67	Extraorbital non-Hodgkin lymphoma	3 cases of dry eye	Total dose: 4 Gy, fractionated at 2 Gy per fraction	39 months.
Meriem Mokhtech (24)	2018	Retrospective analysis	20	43	Pseudotumor of the orbit	10% cases of dry eye	Sixty per cent of patients received 20 Gy, 20 per cent received a dose below 20 Gy, and 20 per cent received a dose above 20 Gy.	96 months.
Giancarlo A. Garcia (25)	2022	Retrospective analysis	48	65.9	Malignant tumor of the orbit	39 cases of dry eye	The median radiotherapy dose was 64.8 Gy.	18.3 months.
Young-Woo Jeon (26)	2018	Retrospective analysis	117	46	Orbital lymphoma	59% cases of dry eye	The median radiotherapy dose was 26Gy.	70 months.
Nan Ma (27)	2023	Retrospective analysis	10	60.1	Orbital tumor	3 cases of dry eye	The number of seeds implanted ranged from 16 to 40.	40-65months.
Sharad Goyal (28)	2008	Retrospective analysis	4	71.5	Orbital lymphoma	1 cases of dry eye	The median radiotherapy dose was 30.6 Gy.	12months.
Alexandra I. Manta (29)	2025	Retrospective analysis	21	62	Orbital lymphoma	14% cases of dry eye	Total dose: 4 Gy, fractionated at 2 Gy per fraction	27months.
Megha Kaushik (30)	2011	Retrospective analysis	67	67	Orbital lymphoma	39% cases of dry eye	The median radiotherapy dose was 30.33 Gy.	36.5 months.
Michael S. Binkley (31)	2015	Retrospective analysis	32	51	Orbital lymphoma	3 cases of dry eye	The median radiotherapy dose was 30.6 Gy.	45.8 months.

### Quality assessment

The (NOS) was employed to assess the quality of cohort studies (**Table 2**). During evaluation, each domain was assigned a specific score, with a maximum total of 9 points. Scores for individual domains could be modified based on the study’s particular circumstances to better suit the assessment of specific research. Studies scoring 7–9 points were deemed high quality, 4–6 points indicated moderate quality, and scores below 4 points were classified as low quality. Information on articles accepted for in-depth analysis was recorded in an Excel spreadsheet, primarily concerning variables considered in the review. GRADE quality ratings for assessing the reliability of evidence are presented in (**Supplementary Table 1**). This study examined the risk of dry eye syndrome following radiotherapy for orbital tumors, excluding other ocular complications. Additionally, studies involving irradiation of the eye for head and neck cancers other than orbital tumors were excluded. Outcomes of interest comprised dry eye syndrome and its grading or severity.

**Table 2:** Quality assessment of included studies using the Newcastle-Ottawa scale (NOS).

Study	Selection1	Selection2	Selection3	Selection4	Comparability	Outcome 1	Outcome 2	Outcome 3	Quality scores
Raffaele Nuzzi2020	1	0	1	1	2	1	1	1	8
NAOKIHASHIMOTO2012	1	0	1	1	2	1	1	1	8
Olga Esik1996	1	0	1	1	2	1	1	1	8
Roshan S. Prabhu2012	1	0	1	1	2	1	1	1	8
Carolina E. Fasola2013	1	0	1	1	2	1	1	1	8
R. Beverly Raney2000	1	0	1	1	2	1	1	1	8
Yasuo Ejima2006	1	0	1	1	2	1	1	1	8
Jayant S. Goda2010	1	0	1	1	2	1	1	1	8
Christian Hoffmann2022	1	0	1	1	2	1	1	1	8
Shubha Tiwari2017	1	1	1	1	2	1	1	1	9
Jayant Sastri Goda2011	1	0	1	1	2	1	1	1	8
PING ZHOU2004	1	0	1	1	2	1	1	1	8
Sanjna Shelukar2022	1	0	1	1	2	1	1	1	8
Meriem Mokhtech2018	1	0	1	1	2	1	1	1	8
Giancarlo A. Garcia2022	1	0	1	1	2	1	1	1	8
Young-Woo Jeon2018	1	0	1	1	2	1	1	1	8
Pereira-Da Silva2023	1	0	1	1	2	1	1	1	8
Quynh-Thu Le2002	1	0	1	1	2	1	1	1	8
Nan Ma 2021	1	0	1	1	2	1	1	1	8
Sharad Goyal 2008	1	0	1	1	2	1	1	1	8
Alexandra I. Manta2024	1	0	1	1	2	1	1	1	8
Megha Kaushik2011	1	0	1	1	2	1	1	1	8
Michael S. Binkley2015	1	0	1	1	2	1	1	1	8

### Statistical analysis

Data extracted using Microsoft Excel were imported into Stata 17 for statistical analysis. The I^2^ statistic was employed to assess heterogeneity among studies. Given the presence of substantial heterogeneity, a random-effects model was utilized to estimate the pooled prevalence of dry eye syndrome. Concurrently, publication bias was evaluated subjectively via funnel plots and objectively through Egger’s test. Furthermore, we explored sources of heterogeneity through subgroup analyses stratified by age, dose, follow-up duration, and tumor subtype. Sensitivity analyses were conducted by sequentially excluding individual studies.

Following consultation among all researchers, several questions regarding the included studies were raised: Is the incidence of dry eye syndrome after radiotherapy for orbital tumors statistically significant? Do factors such as radiation dose, follow-up duration, and orbital tumor classification influence the occurrence of dry eye syndrome?

## Results

We conducted a targeted summary addressing the aforementioned issues. Prior to this, following the methodology outlined, we screened multiple databases separately, identifying a total of 9,209 studies. Through comprehensive retrieval steps and analytical methods, and after consultation among all authors, 23 studies were ultimately selected. PRISMA diagram) illustrates the entire screening process (**Figure 1**). Through detailed retrieval, 9,209 articles were initially screened. First, duplicate articles were excluded. Next, studies involving radiotherapy for head and neck cancers other than orbital tumors were removed, as these were less relevant to our theme. Articles lacking full-text availability, systematic reviews, and meta-analyses were subsequently excluded. Ultimately, 23 articles were included in the review. These were assessed using the NOS scale and were all high-quality studies (**Table 2**).

The 23 studies encompassed 1,602 patients with orbital tumors, all employing retrospective analysis. Extracted data are presented(**Table 1**). The majority of publications appeared within the last two decades, with four studies dating from earlier periods: 1996, 2000, 2002, and 2004. The overwhelming majority of orbital tumor types reported were orbital lymphomas. Only three studies addressed tumors other than orbital lymphoma: two concerning orbital pseudotumors and one on rhabdomyosarcoma. All included studies reported the occurrence of dry eye syndrome. Two publications summarised the grading of post-radiotherapy dry eye syndrome, aiding in establishing further links between tumor radiotherapy and dry eye grading. Studies with only one case and a sample size exceeding five were excluded as incidental (3 studies).

**Figure 2:**
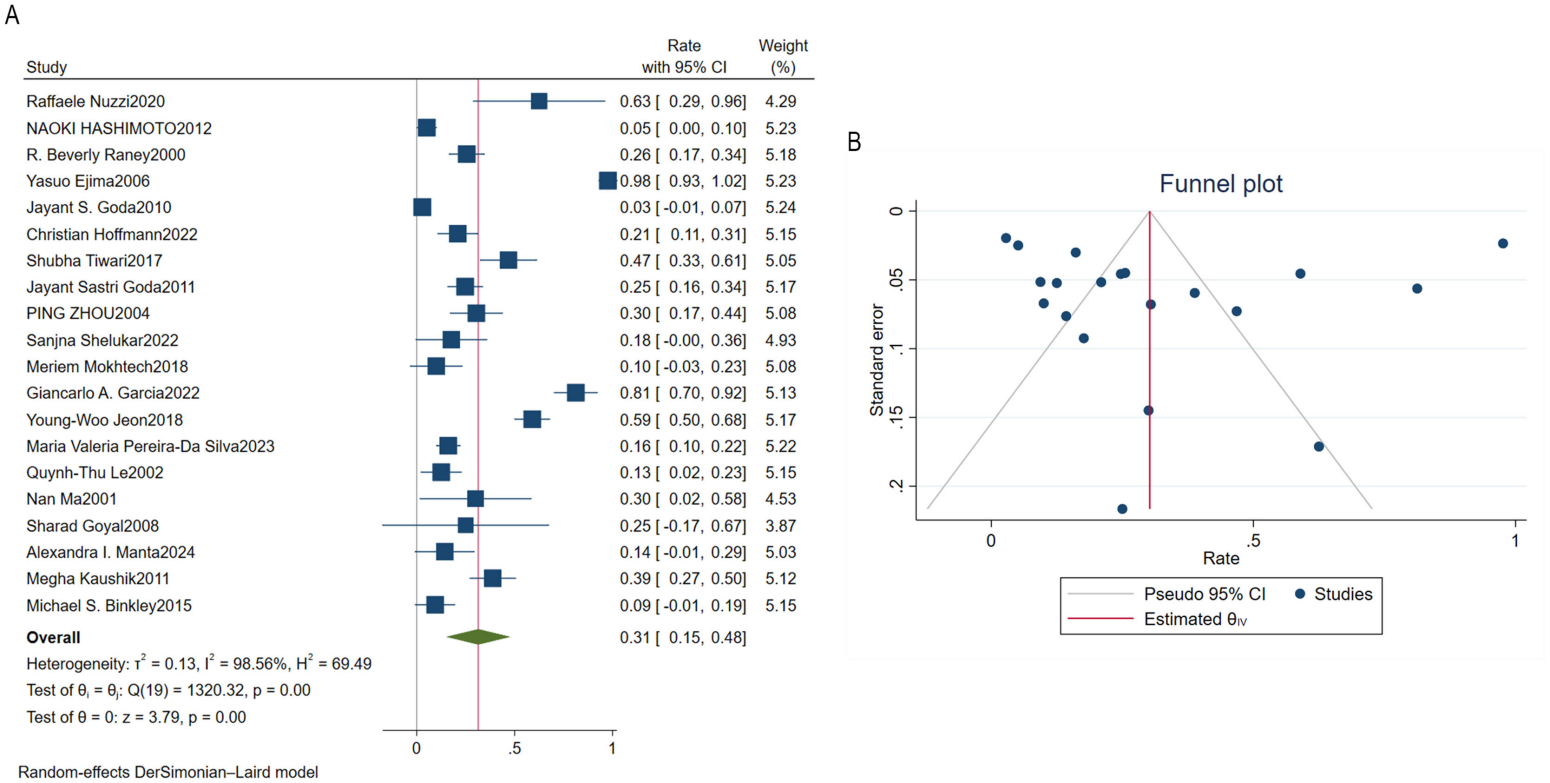
Prevalence of dry eye syndrome following radiotherapy for orbital tumors. **(A)** Forest plot of dry eye syndrome prevalence after radiotherapy for orbital tumors. **(B)** Funnel plot of bias analysis for dry eye syndrome prevalence after radiotherapy for orbital tumors.

Results from 20 studies indicated that the prevalence of dry eye syndrome following radiotherapy for orbital tumors was 31%, with a 95% confidence interval [15%, 48%]. The p-value< 0.01, demonstrating a statistically significant difference between studies (**Figure 2A**). The heterogeneity analysis yielded an I^2^ value of 98.56%. As I^2^ > 50%, a random-effects model was employed. The Egger test (P = 0.8452) and Begg test (P = 0.1630) were conducted for bias assessment. A funnel plot was generated, revealing no significant publication bias (**Figure 2B**).

We subsequently conducted subgroup analyses to investigate the causes of high heterogeneity and the presence of confounding factors (**Table 3**). First, subgroup analysis by age showed a prevalence of 28% [12%, 43%] in those under 60 years old, P< 0.01, and 41% [12%, 71%] in those aged 60 years and above, P = 0.01. Subsequently, subgroup analyses were conducted for total doses below and above 30 Gy. The prevalence rate below 30 Gy was 25% [11%, 38%], P< 0.01, while the rate above 30 Gy was 36% [10%, 62%], P = 0.01. The incidence rate was 40% [16%, 64%] for follow-up durations below 5 years and 19% [8%, 31%] for those above 5 years, with both P < 0.01. Finally, we analyzed the impact of tumor subtype: non-orbital lymphoma 39% [12%, 66%], orbital lymphoma 29% [10%, 48%], with P <0.01 for both (**Supplementary Figure 1-4**). Subgroup analysis revealed that the incidence rate among patients aged 60 years and above (41%) was higher than that among those under 60 years (28%). The incidence of dry eye syndrome was 36% in patients receiving total doses exceeding 30 Gy, compared to 25% in those receiving less than 30 Gy. With extended follow-up, the incidence rate after artificial tear treatment was 19% in patients followed for over five years, lower than the 40% observed in those followed for less than five years. Non-orbital lymphoma patients exhibited a higher incidence of dry eye syndrome than those with orbital lymphoma (39% vs 29%). Overall, the P-values for interaction were all greater than 0.05. The effect sizes across subgroups were consistent in direction, indicating a uniform trend in intervention effectiveness across different subgroups. The influence of subgroup factors on intervention outcomes was minimal.

**Table 3:** Subgroup analysis of dry eye syndrome risk following orbital radiotherapy.

Subgroup	Study count	Effect(95%CI)	P value	I^2^	P for interaction
Year					0.603
<60	8	28%[12%,43%]	P<0.01	92.75%	
≥60	8	41%[12%,71%]	P=0.01	98.03%	
Total dose					0.262
<30Gy	9	25%[11%,38%]	P<0.01	94.52%	
≥30Gy	11	36%[10%,62%]	P=0.01	98.92%	
Follow-up time					0.257
<5 year	12	40%[16%,64%]	P<0.01	97.88%	
≥5 year	8	19%[8%,31%]	P<0.01	95.47%	
Tumor subtype					0.450
Non-orbital lymphoma	5	39%[12%,66%]	P<0.01	95.31%	
Orbital lymphoma	15	29%[10%,48%]	P<0.01	98.77%	

**Figure 3:**
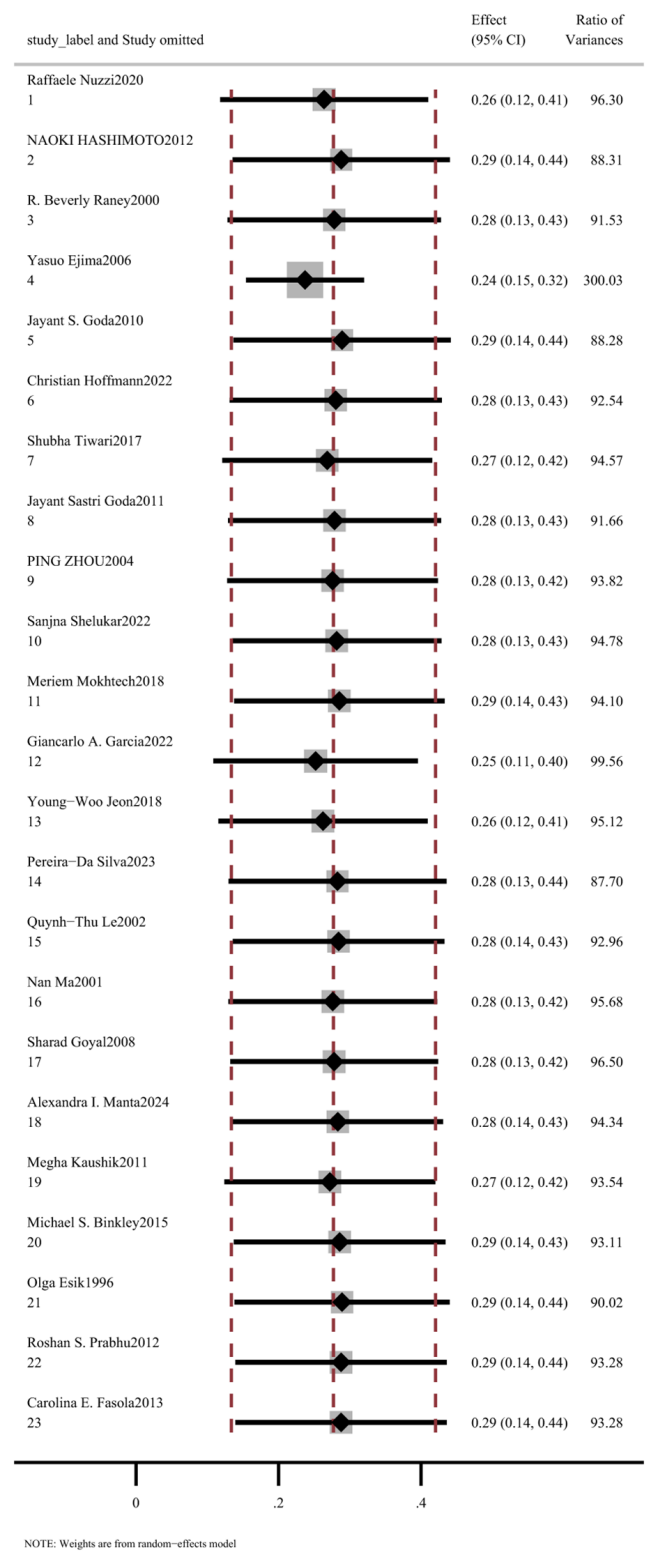
Sensitivity analysis of dry eye syndrome risk following radiotherapy for orbital tumors.

We subsequently conducted a sensitivity analysis by sequentially excluding each study. Overall, the results remained stable in all studies except one (**Figure 3**).

## Discussion

Radiotherapy has been demonstrated as a significant treatment modality for orbital tumors. Compared to surgical intervention, it exhibits greater radiosensitivity, yielding favorable local control and curative outcomes. Studies categorize doses into low-dose groups (4–6 Gy), medium-dose groups (24–30.6 Gy), and high-dose groups (>30.6 Gy). This research indicates no significant impact of differing doses on survival rates. Late-stage toxicities such as dry eye syndrome occurred significantly less frequently in the low-dose group. The lacrimal gland and meibomian glands are highly radiosensitive ocular tissues. Irradiation of the former causes loss of secretory function, impairing the maintenance of normal ocular moisture and predisposing patients to dry eye syndrome. The latter, by hindering the evaporation of tears, exacerbates this condition (9).

While the impact of age and radiation dose on tear film stability is well-documented in head and neck cancers, our findings define the specific risk thresholds for orbital tumor patients. For instance, the incidence significantly escalates when the dose≥30 Gy. This provides a more precise parameter for radiation oncologists to balance tumor control and lacrimal apparatus sparing. Our subgroup analysis revealed that patients with non-orbital lymphoma experienced higher DES incidence (39%) than those with orbital lymphoma (29%). This disparity might be attributed to the larger radiation fields often required for non-orbital involvement, which inadvertently encompass more of the accessory lacrimal glands and meibomian glands.

### Implication

Results from 20 studies indicated a prevalence of dry eye syndrome following radiotherapy for orbital tumors of 31%, with a 95% confidence interval [15%, 48%]. The p-value was less than 0.01, demonstrating a statistically significant difference between studies. Heterogeneity analysis yielded an I^2^ of 98.56%. As I^2^ > 50%, a random-effects model was employed. Egger’s test (P = 0.8452) and Begg’s test (P = 0.1630) were conducted for bias assessment. A funnel plot revealed no evident publication bias. Subgroup analysis revealed that age, follow-up duration, dose, and tumor subtype were consistent with the direction of prevalence, with interaction P-values between groups all >0.05. Sensitivity analysis demonstrated robust results. In our study, the pooled prevalence of DES was 31%, with significant statistical heterogeneity ( ${\text{I}}^{2}=98.56\%$). This high heterogeneity is consistent with other prevalence meta-analyses in radiotherapy. Clinical heterogeneity may stem from the evolution of radiotherapy techniques from 2D-RT in the 1990s to modern IMRT and proton therapy, as well as the varied diagnostic criteria for DES used across the included studies. This study constitutes a meta-analysis of single-group rates, exhibiting considerable heterogeneity, which represents one of its limitations.

The dose-response curve for radiotherapy-induced dry eye syndrome is also crucial, including the time-response curve. Subgroup analysis revealed that the incidence rate among patients aged 60 years and above (41%) was higher than that among those under 60 years (28%). The incidence rate of dry eye syndrome was 36% in patients receiving a total dose exceeding 30 Gy, compared to 25% in those receiving less than 30 Gy. With prolonged follow-up, the incidence rate after five years of artificial tear treatment was 19%, lower than the 40% observed within five years. Non-orbital lymphoma exhibited a higher incidence of dry eye syndrome than orbital lymphoma (39% vs 29%). This study differs from others in statistically quantifying the incidence of dry eye syndrome (32). Despite this variability, our subgroup analyses (**Table 3**) confirmed that higher doses (>30 Gy) and older age remain consistent risk factors. Furthermore, leave-one-out sensitivity analysis demonstrated that the 31% estimate is robust and not driven by any single outlier study, ensuring the reliability of our clinical conclusions. It further determined that factors such as radiation dose, age, tumor subtype, and follow-up duration do not influence the occurrence of dry eye syndrome.

Decreased tissue repair capacity: Lacrimal glands and meibomian glands comprise acinar cells and ductal epithelial cells. Ageing leads to diminished stem cell activity, weakened cellular regeneration, and reduced efficiency of DNA repair mechanisms. DNA damage and apoptosis induced by radiotherapy prove more challenging to repair in elderly patients. Lacrimal glands exposed to doses exceeding 30 Gy may sustain irreversible damage to secretory function. This correlates with the higher incidence of dry eye in the ≥30 Gy group (36% vs 25%) observed in our study results. Tumor type confounding: Although lymphoma predominated, the cohort included pseudotumours, rhabdomyosarcomas, and other malignancies. Treatment doses and field design varied significantly across tumor types. Variations in radiotherapy protocols: Total doses (4 Gy to 64.8 Gy), fractionation schedules, and the inclusion of adjuvant surgery or chemotherapy were inconsistent.

### Limitations

The majority of analyses included in this study concerned the incidence and prevalence of dry eye syndrome, with only a few studies mentioning dry eye grading, Schirmer’s test, or BUT or OSDI questionnaire scores. Among all patients, the incidence of DES was 76% (95% confidence interval: 0.64–0.88), affecting 38 of 50 eyes, with a mean latency period of 45.67 ± 21.63 months. Within this cohort, the lymphoma group receiving 24 Gy irradiation (mean maximum ocular dose: 17.86 ± 9.49 Gy) exhibited a DES incidence of 66.67% (95% confidence interval: 0.40–0.93), whereas the lymphoma group receiving 4 Gy irradiation (mean maximum ocular dose: 2.31 ± 2.20 Gy) exhibited a rate of 50% (95% confidence interval: 0.01–0.99) (9).These parameters facilitate a more nuanced interpretation. Due to the limited granularity of data in the included literature, we were unable to perform meta-analyses on DES grading (severity) or specific diagnostic parameters like BUT and Schirmer’s test. Future research should transition from reporting simple incidence rates to more nuanced assessments of ocular surface quality of life post-radiotherapy. Finally, a limitation is the high heterogeneity. While we explored factors like dose and age, other critical parameters—such as the mean dose to the lacrimal gland or meibomian gland sparing—were frequently not reported in the original literature. This lack of granular data limited our ability to fully resolve the sources of heterogeneity.

### Future research

Future research should focus on grading dry eye syndrome following radiotherapy, extending beyond Schirmer’s test, BUT, or OSDI questionnaire scores. This would enable a more detailed explanation of dry eye syndrome development, facilitating adjustments to radiotherapy protocols. One study compared 40 patients with conjunctival or orbital lymphoma receiving an average dose of 37.4 Gy with 60 healthy controls, assessing Schirmer’s test, (BUT), and OSDI questionnaire scores. Results demonstrated that the irradiation group exhibited significantly lower mean Schirmer test values (9.2 ± 5.1 mm) and mean BUT values (4.2 ± 2.5 seconds) compared to the control group (12.3 ± 5.2 mm and 6.4 ± 2.6 seconds, respectively), while OSDI questionnaire scores were significantly higher than the control group (48.1 ± 21.4 vs. 6.2 ± 4.4) (33–36).

### Conclusion

Radiotherapy for orbital tumors significantly elevates the risk of DES. Clinical attention should be directed toward high-risk subgroups, including elderly patients and those receiving higher radiation doses, to facilitate early intervention and improve ocular health outcomes.

## Data Availability

The original contributions presented in the study are included in the article/**Supplementary Material**. Further inquiries can be directed to the corresponding author.
