# Supplementary material for: Risk of dry eye syndrome after radiotherapy for orbital tumors: a systematic review and meta-analysis
Source: Front Oncol. 2026 Mar 25;16:1786414. doi: 10.3389/fonc.2026.1786414 (PMC13056652; doi:10.3389/fonc.2026.1786414)
Supplement: Supplementary file 1 [file Table1.docx]

Supplemental figure1: Subgroup analysis for age


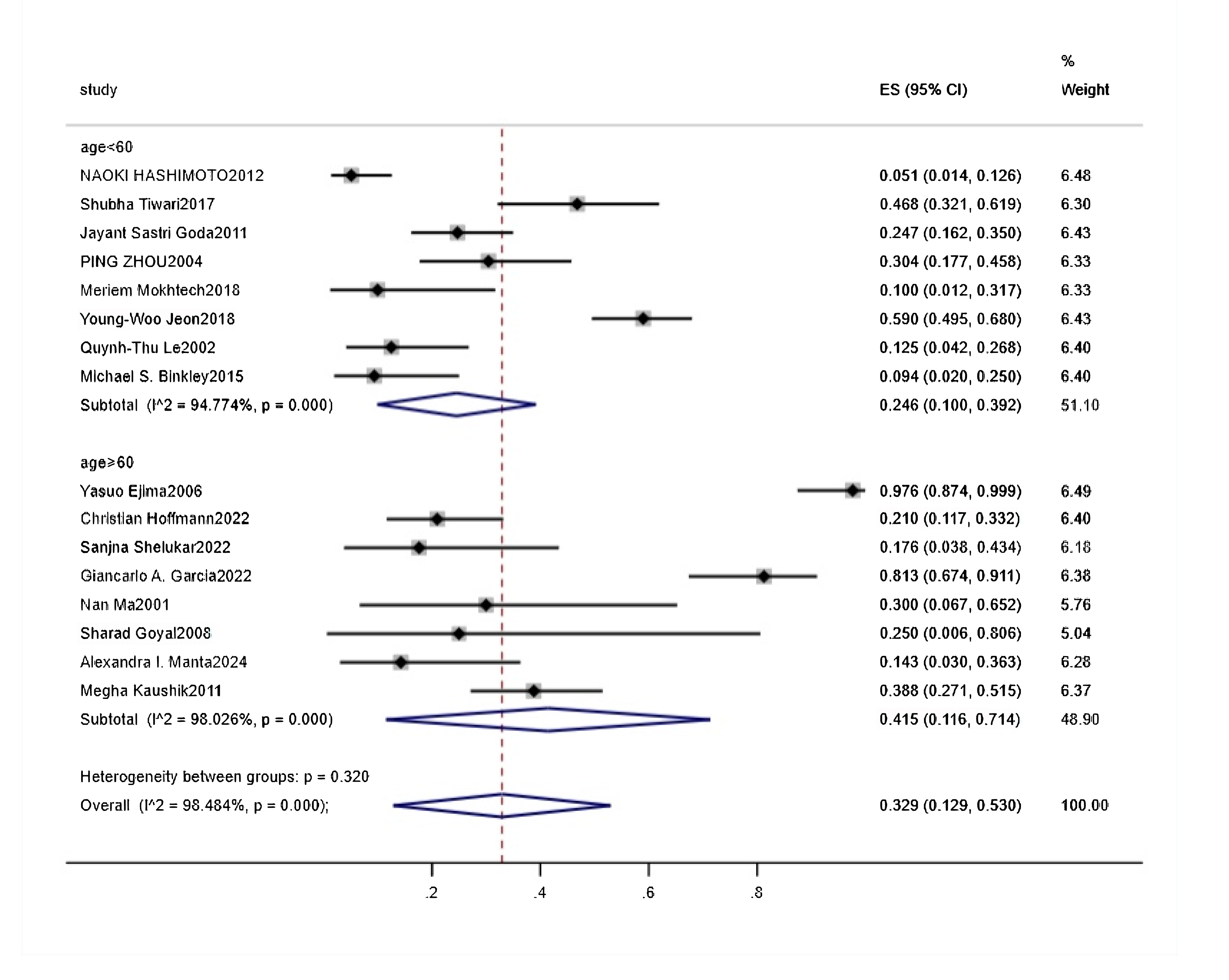


Supplemental figure2: Subgroup analysis for dosage

Supplemental figure2: Subgroup analysis for dose


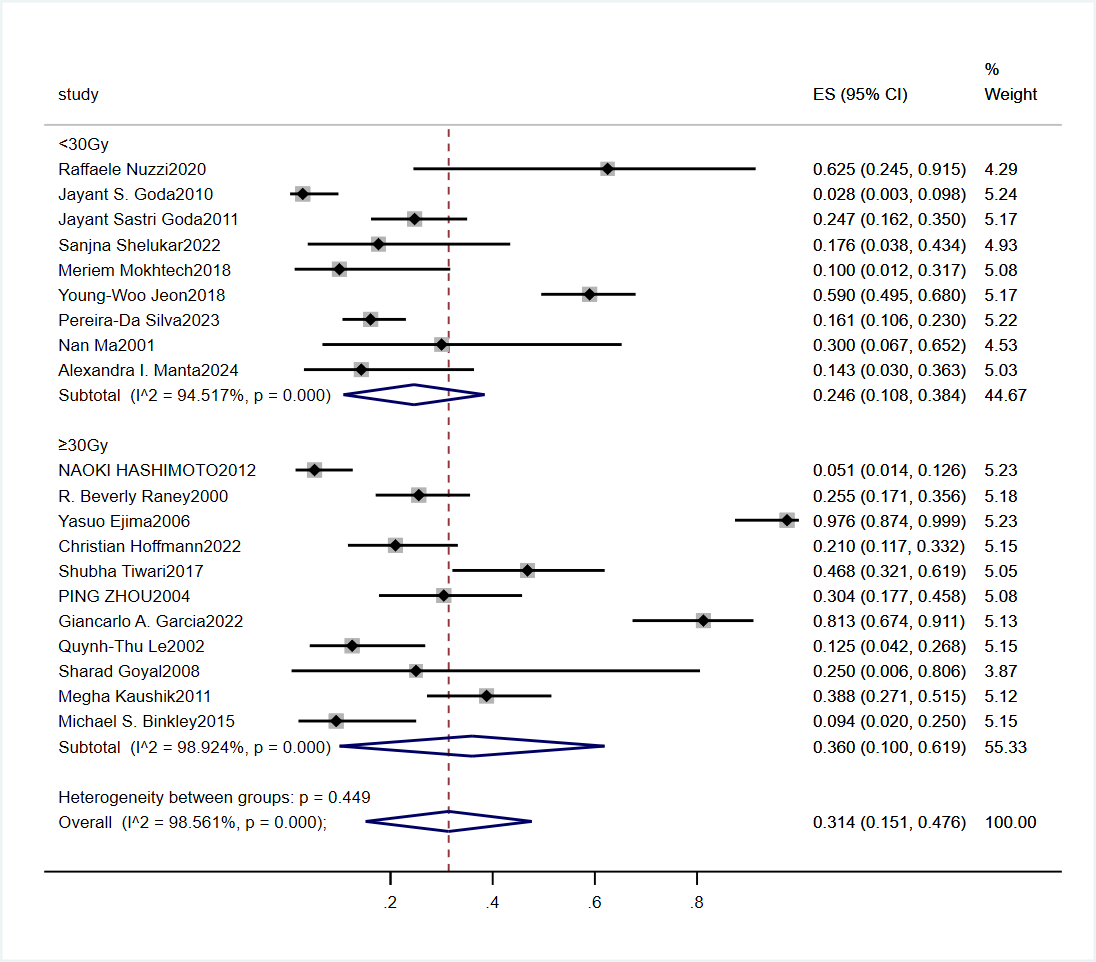


Supplemental figure3: Subgroup analysis for follow-up duration


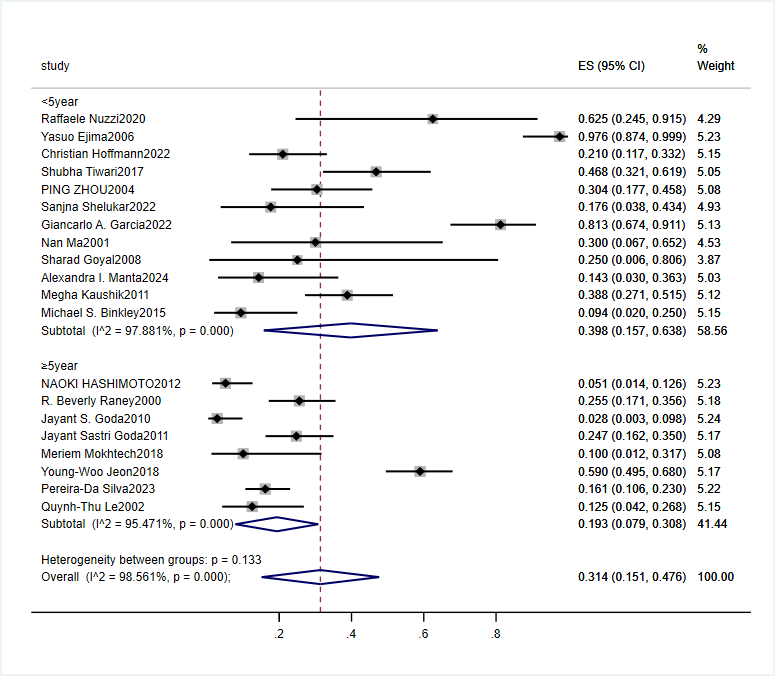


Supplemental figure4: Subgroup analysis for tumour subtypes


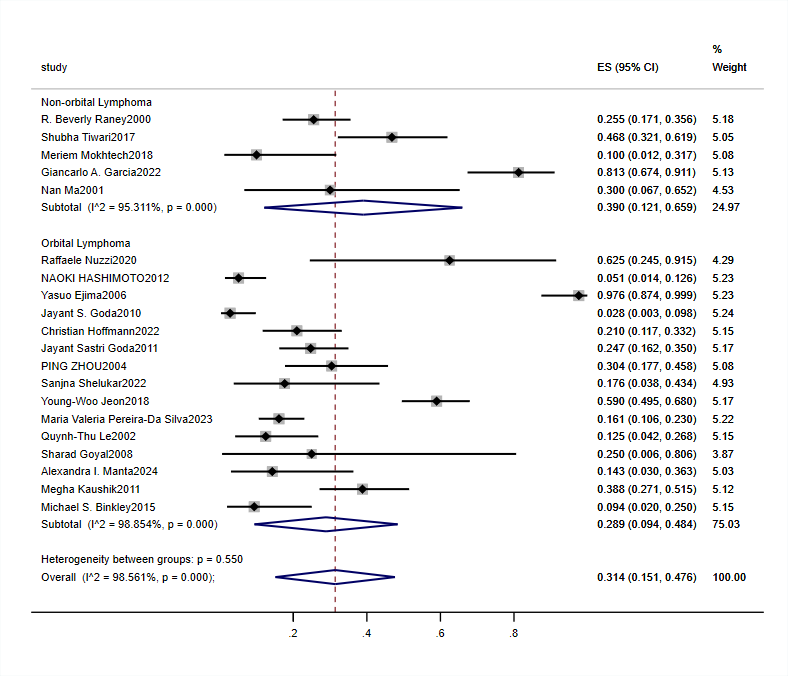

 Supplemental table1: GRADE assessment
